# Supplementary material for: Is a higher altitude associated with shorter survival among at-risk neonates?
Source: PLoS One. 2021 Jul 14;16(7):e0253413. doi: 10.1371/journal.pone.0253413 (PMC8279317; doi:10.1371/journal.pone.0253413)
Supplement: S1 File — (DOCX) [file pone.0253413.s002.docx]

### **S1 File. Material and methods complete version.**

### Design:

We performed a nationwide retrospective analysis of registered deaths in Ecuador of all neonates who died ≤28 days of life and were registered in 126 public and private health care facilities into the Surveillance System of Neonatal Mortality of the Public Health Ministry of Ecuador, from January 2014 to September 2017. We excluded from the analysis neonates who died in the prehospital setting, because of lack of information about the altitude of their health care attendance (see the diagram in S1 Fig.).

### Population and database:

All registered neonatal deaths were included in this study. The epidemiologist in each health care facility systematically reports a form to this system, describing neonatal deaths up to 24 hours after they happened. Despite the database includes perinatal information from all registered deceased neonates –at-risk neonates–, it did not include information on maternal education and other contextual variables at the individual level.

### Main outcome:

We performed a survival analysis by setting the neonatal survival time, as measured by days, as the primary outcome. Then, we calculated hazard ratios (HR) using Cox’s proportional hazards models, and we compared the survival time across altitude strata.[1] Despite information about non-deceased neonates being unavailable, the survival analysis gave us the probability of event occurrence (i.e., neonatal death) per each altitude stratum.

### Process of building the database

The database is built based on the information collected from all hospitals in the Public Network of Health Establishments (made up of all public hospitals and its associates) and the Private Network of Health Establishments, including all Specialty Hospitals of Gynaecology and Obstetrics, as well as any hospital that attends births. The database is built from systematic reports from the health establishments whenever there is a newborn death (≤28 days of life). The reports are sent in through a pre-established form by using a unique username and password for each establishment. This username is assigned by the system manager (DNVE).

### Actors of the health system which are obligated to report neonatal deaths

All public and private health establishments in the country are obligated to report any neonatal death in the first 24 hours after the event.

### Established criteria for reporting neonatal deaths

There are two pre-established forms and two ways to notify neonatal deaths in the system. The first one is a simplified report where all neonatal deaths are reported. The second one registers both the information from the simplified report and other additional information, including criteria that show preventability. This data includes some anonymized information from the mother.

### Verifying information

There is an epidemiologist responsible for each political-administrative zone. This epidemiologist has a username and password that gives him/her access to all the information reported in the designated area. The epidemiologist is responsible for reviewing the information and detecting incongruences, by verifying with those who report it.

### Main explanatory variable:

The altitude of the health care center in which neonates were attended was the main explanatory variable. Three independent researchers used information from the GeoSalud 3.6.0 web viewer [www.geosalud.com](http://www.geosalud.com) and the computer program [www.googlearth.com](http://www.googlearth.com) for verifying the altitude of each facility. To categorize the altitude, we built a histogram to evaluate the possibility of dividing altitude categories by centiles; but, due to the irregular Ecuadorian geography, the particular distribution of neonatal deaths in each altitude interval made the categorization in tertiles or quartiles very difficult (Figure 2S); although a continuous analysis of the altitude data could reduce unintended biases and it would be more useful for comparison of estimates with other studies, we decided to only keep a categorical analysis, due to the important overdispersion of the data (Figure 3S).

In order to perform a proper categorization of the altitude we considered several geographic factors. Ecuador has four regions, each with different altitude and specific distribution of the population. The coast region and the Galapagos Islands region are located at an altitude that varies among 0–500 m. The highlands include the Andean mountains, located at beyond 1501 m. The Amazon region is located between 501–1500 m [2]. The diversity of climates and altitude levels, the political division of the country, and the limited access to basic services, determine a particular configuration of the population´s density. Consequently, regarding the evaluated health outcome, distinct studies established different altitude clusters. For example, a study of the prevalence of metabolic syndrome in Ecuador, used the above-mentioned categories [2]. Contrary, a study about congenital heart disease in Ecuador, determined three altitude clusters according to CHD prevalence: 2500 to 2750 m; 2751 to 3000 m; and 3001 and 3264 m [3]. The last ones are similar to our altitude clusters, but noticeably they do not include low altitude clusters since the prevalence of CHD is not significant in those regions. Thus, the patterns of altitude categorization that we used, are according to our research objective.

### Other covariates

Given that there are several potential confounders in the association between altitude and neonatal survival time, variables were divided into two types: *(i)* individual covariates: birth weight, gestational age at birth, small for gestational age –neonates whose birth weight was less than the 10th percentile for gestational age–,[4] using the Intergrowth equations,[5] Apgar scale at 5 minutes, type of delivery, and comorbidities; and, *(ii)* contextual covariates: administrative planning areas, type of health care facility, and level of care, 2014 to 2016 Gini coefficient at the province level, province of the habitual residence of the mother, as well as province of neonatal health care, to identify neonates who died outside their mother’s usual residence province, and rural-urban health care facility.

### Statistical analyses:

Despite the analyses being performed using the whole database of Ecuadorian neonatal mortality from 2014 to 2017, we corroborated that a sample size of no less than 100 observations per altitude stratum was enough to allow meaningful comparative survival analyses of the categories chosen.[6].

### Sample size considerations

The analyses were performed using the whole database of Ecuadorian neonatal mortality from 2014 to 2017, and we corroborated, *a posteriori*, that a sample size of no less than 100 observations per altitude stratum was enough to allow meaningful comparative mortality risk analyses of the categories chosen, by using a sample size calculation for survival analysis[6] (http://www.sample-size.net/sample-size-survival-analysis/). Considering: *(i)* a two-tailed alpha of 0.05, *(ii)* a β of 0.20, *(iii)* a proportion of 0.95 of subjects in the unexposed to altitude group (*i.e.,* <80 m) and a proportion of 0.05 of subjects in the exposed group (*i.e.,* ≥80 m), *(iv)* a relative hazard of at least 1.40, *(v)* a baseline event rate for the unexposed group of 0.16 deaths per patients-day, *(vi)* a median survival time in unexposed of 4 days, and, *(vii)* a planned average length of follow-up of 15 days; we calculated a sample of at least 100 patients in the exposed stratum and, at least, 1902 patients in the unexposed stratum; which represents, a total n of no less than 2002 patients. Finally, the population studied was 3016 patients, with no less than 156 patients in the smallest altitude stratum. Finally, we analyzed 3016 deceased neonates, with 156 patients in the smallest altitude stratum.

Descriptive statistics were performed using percentages for categorical variables and median and P25 to P75 for discrete variables. To assess the differences of each individual and contextual variable across altitude categories, we performed: *(i)* Kruskal Wallis tests for assessing differences of Apgar score at 5 minutes and gestational age; *(ii)* ANOVA for assessing differences in birth weight *(iii)* Chi2 tests for assessing differences of small for gestational age, type of delivery, comorbidities, tertiles of Gini at the provincial level, type of health care center, level of care, neonatal deaths outside their mother’s usual residence province, and rural-urban health care facility; and, *(iv)* log-rank test for equality of survivor functions for assessing differences of neonatal survival time, as measured by days, –the main outcome–.

Then, we estimated crude and adjusted HR per each stratum of altitude.[1] In that sense, we built multivariate Cox proportional hazards models, to evaluate the independent association between altitude and neonatal survival time among at-risk neonates.

We built a saturated model that included all individual covariates and, based on the researchers' expertise, we selected the covariates that were retained in the parsimonious model (S1 Table), which shows the variables that were excluded from the saturated model, as well as the estimates resulting from the parsimonious model. Hazard ratios were estimated. The 95% confidence intervals (95% CI) of the HR and their corresponding p-values were obtained. Once the parsimonious model was obtained, we compared both models and chose the “final” model, according to its level of significance from the likelihood ratio test.

To assess the effects from contextual variables on mortality, we estimated HR by mixed-effects Cox proportional hazards models, considering contextual variables in model 1: administrative planning areas, because of potential differences in quality of care across those zones, type of health care facility (*i.e.*, public or private) because of differential quality of care across the type of care, and level of care (*i.e.*, primary, secondary or tertiary care) because of differential resources on neonatal health care, in model 2: administrative planning areas, and level of care; and in model 3: level of care, only.

We performed several secondary analyses to assess the sensitivity of our estimates to our assumptions regarding biases, as well as to test for model misspecification. First, considering that differential treatment for individual causes of death (for example, asphyxia, infectious diseases, etc.) could affect the estimates, we ran final model excluding neonates who died from *(i)* asphyxia related disorders, *(ii)* congenital malformations, *(iii)* prematurity related disorders, *(iv)* infectious disorders. Second, in order to verify estimations did not change significantly between the highest and lowest risk of adverse outcomes, we excluded those neonates with <5 points and those with ≥7 Apgar score at 5 minutes. Third, knowing that migration from the residence where the pregnancy occurred could modify the estimates, we tested that, excluding neonates who died in a different province than their mother's habitual residence. Fourth, we built four binomial secondary outcomes of survival: death before 24, 48, 72 hours, and before 7 days of life and ran four mixed-effects multivariate logistic regression models to estimate adjusted odds ratios covariates used in model 1. Fifth, regarding the fact that the database is a death registry –and all neonates die–, it represents a right truncated database. Right truncation occurs when patients who experience the event are selectively included, and hence, survivors are not selected for the study. Despite potential bias caused by truncation exists, several studies have been used right truncated databases for survival analysis[7–9], addressing such potential source of bias by using Inverse Probability Weighted (IPW) Cox Regression.[7] In that sense, we performed an IPW analysis and calculated the estimates of survival in days of life across altitude strata by artificial cut-offs at 15, 17, 20, and 23 days of the retrospective follow-up.

Furtherly, to assess the effects from other contextual variables like Gini coefficient at province level and rural-urban health care, we estimated HR from mixed-effects multivariate Cox proportional models, in this way: fixed effects for next individual variables: gestational age, birth weight, Apgar scale at 5 minutes, comorbidities, and random effects for contextual variables: *(i)* Gini coefficient at the provincial level, type of health care facility, and level of care in Model 4; *(ii)* Gini coefficient at the provincial level in Model 5; *(iii)* rural-urban health care facility, type of health care facility and level of care in Model 6; and rural-urban health care facility in Model 7.

Given the small number of missing data (S2 Table), we employed complete case analysis in estimating statistical associations. We considered that there were statistically significant differences when the p-value<0.05. Analyses were performed with Stata 14.2 (Statistical Software Stata: Release 14.2 College Station, TX: StataCorp LP).

## This study was part of the “Score Bebé” project and was conducted with the Research Ethics Committee in Human Beings (CEISH) of the Pontificia Universidad Católica del Ecuador approval (code number: 2018-09-EO).

## References of the material and methods complete version.

1. Bewick V, Cheek L, Ball J. Statistics review 12: Survival analysis. Crit Care. 2004;8(5):389–94.

2. Pérez-Galarza J, Baldeón L, Franco OH, Muka T, Drexhage HA, Voortman T, et al. Prevalence of overweight and metabolic syndrome, and associated sociodemographic factors among adult Ecuadorian populations: the ENSANUT-ECU study. J Endocrinol Invest [Internet]. 2021;44(1):63–74. Available from: https://doi.org/10.1007/s40618-020-01267-9

3. González-Andrade F. High Altitude as a Cause of Congenital Heart Defects: A Medical Hypothesis Rediscovered in Ecuador. High Alt Med Biol. 2020;21(2):126–34.

4. American College of Obstetricians. ACOG Practice bulletin no. 134: fetal growth restriction. Obstet Gynecol. 2013;121(5):1122–33.

5. Villar J, Giuliani F, Bhutta ZA, Bertino E, Ohuma EO, Ismail LC, et al. Postnatal growth standards for preterm infants: the Preterm Postnatal Follow-up Study of the INTERGROWTH-21(st) Project. Lancet Glob Heal. 2015;3(11):e681-91.

6. Schoenfeld DA. Sample-Size Formula for the Proportional-Hazards Regression Model. Biometrics. 1983;39(2):499.

7. Mandel M. Inverse Probability Weighted Cox Regression for Doubly Truncated Data. Biometrics. 2018;74(2):481–7.

8. Conner SC, Sullivan LM, Benjamin EJ, LaValley MP, Galea S, Trinquart L. Adjusted restricted mean survival times in observational studies. Stat Med. 2019;38(20):3832–60.

9. Lagakos SW, Barraj LM, Gruttola V De. Nonparametric analysis of truncated survival data, with application to aids. Biometrika. 1988;75(3):515–23.
