# Supplementary figures and images for: Is a higher altitude associated with shorter survival among at-risk neonates?
Source: PLoS One. 2021 Jul 14;16(7):e0253413. doi: 10.1371/journal.pone.0253413 (PMC8279317; doi:10.1371/journal.pone.0253413)

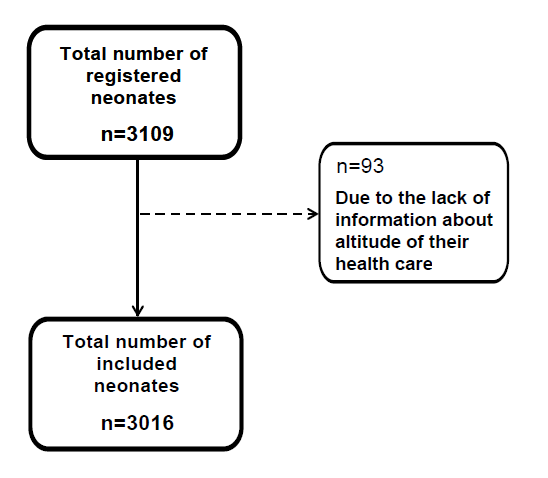

Supplement: S1 Fig — (TIF) [file pone.0253413.s003.tif]

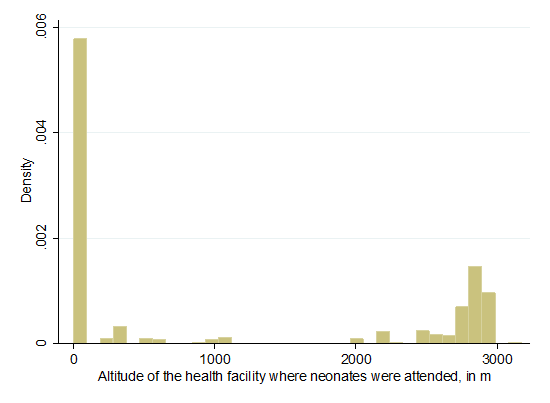

Supplement: S2 Fig — (TIF) [file pone.0253413.s004.tif]

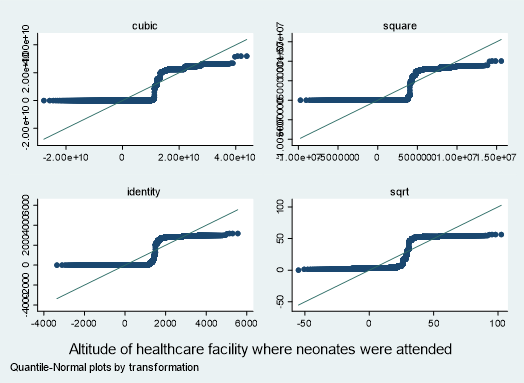

Supplement: S3 Fig — (TIF) [file pone.0253413.s005.tif]
