# Supplementary material for: Is a higher altitude associated with shorter survival among at-risk neonates?
Source: PLoS One. 2021 Jul 14;16(7):e0253413. doi: 10.1371/journal.pone.0253413 (PMC8279317; doi:10.1371/journal.pone.0253413)
Supplement: S1 Table — (DOCX) [file pone.0253413.s006.docx]

## S1 Table.- Crude and adjusted neonatal mortality adjusted hazard ratios per each altitude stratum according to mixed-effects multivariate Cox proportional hazards models.

| **Altitude of the health facility where neonates were attended** | **n (%)** | **Adjusted hazard ratios** | | | |  |
| --- | --- | --- | --- | --- | --- | --- |
|  |  | **Saturated Model ^a^**  **(95% CI)** | ***p-value*** | **Parsimonious model ^b^**  **(95% CI)** | ***p-value*** | |
| *0 to <80 m (ref.)* | 1625(54) | 1 | - | 1 | - | |
| *≥80 to <2500 m* | 405 (13) | 1.19 (0.99 to 1.43) | 0.06 | 1.20 (1.01 to 1.44) | 0.03 | |
| *≥2500 to <2750 m* | 156 (5) | 1.43 (1.03 to 1.99) | 0.03 | 1.32 (0.97 to 1.79) | 0.07 | |
| *≥2750 m* | 830 (28) | 1.46 (1.13 to 1.89) | <0.01 | 1.37 (1.08 to 1.75) | 0.01 | |
| *p for trend* | - | 1.13 (1.04 to 1.23) | <0.01 | 1.11 (1.03 to 1.20) | 0.01 | |
| ^a^ Mixed-effects multivariate Cox proportional hazards model adjusted by next individual variables: gestational age, birth weight, small for gestational age, type of delivery, Apgar scale at five minutes, and comorbidities; and random effects for contextual variables: administrative planning areas, type of health care facility, and level of care.  ^b^ Mixed-effects multivariate Cox proportional hazards model adjusted by next individual variables: gestational age, birth weight, Apgar scale at five minutes, and comorbidities; and random effects for contextual variables: administrative planning areas, type of health care facility, and level of care. | | | | | |  |
