# Supplementary material for: Is a higher altitude associated with shorter survival among at-risk neonates?
Source: PLoS One. 2021 Jul 14;16(7):e0253413. doi: 10.1371/journal.pone.0253413 (PMC8279317; doi:10.1371/journal.pone.0253413)
Supplement: S2 Table — (DOCX) [file pone.0253413.s007.docx]

## S2 Table. – Individual and contextual variables of the study population across categories of altitude of the health facility where neonates were attended.

| **Individual and contextual variables ^a^** | **Altitude of the health facility where neonates were attended**  **Total n=3016** | | | | |
| --- | --- | --- | --- | --- | --- |
|  | **<80 m**  **(n=1625)** | **≥80 to <2500 m**  **(n=405)** | **≥2500 to <2750 m**  **(n=156)** | **≥2750 m**  **(n=830)** | **Missing data**  **n (%)** |
| **Individual variables ^b^** |  |  |  |  |  |
| Gestational age in weeks, median (P25 to P75) | 31 (27 to 36) | 33 (28 to 38) | 31 (26 to 35) | 32 (27 to 36) | 20 (<1) |
| Birth weight in g, median (P25 to P50) | 1633 (915) | 1875 (976) | 1556 (878) | 1634 (908) | 18 (<1) |
| Small for gestational age, n (%) | 231 (15) | 66 (17) | 28 (19) | 120 (15) | 143 (5) |
| Apgar score at five minutes, median (P25 to P75) | 6 (4 to 7) | 7 (5 to 9) | 7 (4 to 9) | 8 (5 to 9) | 123 (4) |
| Type of delivery |  |  |  |  |  |
| *C-section, n (%)* | 1025 (64) | 192 (48) | 52 (34) | 451 (55) |  |
| *Vaginal delivery, n (%)* | 509 (32) | 187 (47) | 81 (53) | 311 (38) | 38 (<1) |
| *Dystocic delivery, n (%)* | 66 (4) | 22 (5) | 20 (13) | 62 (8) |  |
| Comorbidities |  |  |  |  |  |
| *Asphyxia related disorders, n (%)* | 418 (26) | 123 (30) | 33 (21) | 152 (18) | 0 (0) |
| *Malformations, n (%)* | 371 (23) | 92 (23) | 29 (19) | 206 (25) |  |
| *Prematurity related disorders, n (%)* | 473 (29) | 83 (21) | 60 (38) | 288 (35) |  |
| *Infectious diseases, n (%)* | 307 (19) | 93 (23) | 29 (19) | 147 (18) |  |
| *Other non-previously classified, n (%)* | 56 (4) | 14 (4) | 5 (3) | 37 (4) |  |
| Neonatal time-to-death in days, P50 (P25 to P75) | 2.4 (0.6 to 8.0) | 2.2 (0.8 to 6.1) | 1.7 (0.3 to 5.0) | 2.4 (06 to 8.1) | 0 (0) |
| **Contextual variables ^b^** |  |  |  |  |  |
| 2014 to 2016 GINI coefficient, mean (SD) | 0.44 (0. 03) | 0.49 (0.05) | 0.48 (0.02) | 0.47 (0.02) | 48 (2) |
| *First tertile,* *<0.426, n (%)* | 1087 (68) | 16 (4) | 0 | 1 (<1) |  |
| *Second tertile, >0.426 to 0.464, n (%)* | 315 (19) | 144 (38) | 84 (54) | 640 (78) |  |
| *Third tertile, >0.464, n (%)* | 207 (13) | 225 (58) | 72 (46) | 177 (22) |  |
| Type of health care center |  |  |  |  |  |
| *Private medical care, n (%)* | 721 (44) | 26 (6) | 0 (0) | 62 (7) | 0 (0) |
| *Public medical care, n (%)* | 904 (55) | 379 (94) | 156 (100) | 768 (92) |  |
| Level of care |  |  |  |  |  |
| *Primary care unit, n (%)* | 8 (<1) | 13 (3) | 5 (3) | 27 (3) | 13 (<1) |
| *Secondary care unit, n (%)* | 507 (31) | 384 (96) | 150 (97) | 282 (34) |  |
| *Tertiary care unit, n (%)* | 1107 (68) | 3 (1) | 0 (0) | 517 (63) |  |
| Neonatal deaths outside their mother’s usual residence province |  |  |  |  |  |
| *Yes, n (%)* | 304 (19) | 56 (14) | 6 (4) | 171 (21) | 0 (0) |
| *No, n (%)* | 1321 (82) | 349 (86) | 150 (96) | 659 (79) |  |
| Rural-urban health care facility |  |  |  |  |  |
| *Rural, n (%)* | 511 (59) | 151 (37) | 68 (44) | 135 (16) | 0 (0) |
| *Urban, n (%)* | 1114 (52) | 254 (63) | 88 (56) | 695 (84) |  |
| **^a^** Except for the percentage of Small for Gestational Age, there were significant differences of the other variables across altitude categories (p-values <0.01, by Chi2, ANOVA, Kruskal Wallis, and Log-rank test for equality of survivor functions, as appropriate. See main text for details).  **^b^** All individual and contextual variables were significantly associated with time-to-death among at-risk neonates. | | | | | |
