# Supplementary material for: Is a higher altitude associated with shorter survival among at-risk neonates?
Source: PLoS One. 2021 Jul 14;16(7):e0253413. doi: 10.1371/journal.pone.0253413 (PMC8279317; doi:10.1371/journal.pone.0253413)
Supplement: S3 Table — (DOCX) [file pone.0253413.s008.docx]

## S3 Table.- Neonatal mortality adjusted hazard ratios per each altitude stratum according to three mixed-effects multivariate Cox proportional hazards models.

| **Altitude of the health facility where neonates were attended** | **n (%)** | **Adjusted hazard ratio ^a^**  **(95% CI)** | ***p-value*** |
| --- | --- | --- | --- |
| **Model 1** |  |  |  |
| *0 to <80 m (ref.)* | 1625(54) | 1 | - |
| *≥80 to <2500 m* | 405 (13) | 1.20 (1.01 to 1.44) | 0.03 |
| *≥2500 to <2750 m* | 156 (5) | 1.32 (0.97 to 1.79) | 0.07 |
| *≥2750 m* | 830 (28) | 1.37 (1.08 to 1.75) | 0.01 |
| *p for trend* | - | 1.11 (1.03 to 1.20) | 0.01 |
| **Model 2** |  |  |  |
| *0 to <80 m (ref.)* | 1625(54) | 1 | - |
| *≥80 to <2500 m* | 405 (13) | 1.23 (1.03 to 1.47) | 0.02 |
| *≥2500 to <2750 m* | 156 (5) | 1.25 (0.93 to 1.69) | 0.13 |
| *≥2750 m* | 830 (28) | 1.32 (1.04 to 1.66) | 0.02 |
| *p for trend* | - | 1.11 (1.03 to 1.20) | 0.01 |
| **Model 3** |  |  |  |
| *0 to <80 m (ref.)* | 1625(54) | 1 | - |
| *≥80 to <2500 m* | 405 (13) | 1.19 (1.00 to 1.42) | 0.05 |
| *≥2500 to <2750 m* | 156 (5) | 1.32 (0.96 to 1.81) | 0.08 |
| *≥2750 m* | 830 (28) | 1.41 (1.10 to 1.82) | <0.01 |
| *p for trend* | - | 1.13 (1.04 to 1.22) | <0.01 |
| ^a^ Estimated hazard ratios from mixed-effects multivariate Cox proportional models. All models (1. 2 and 3) estimated fixed effects for the next individual variables: gestational age. birth weight. Apgar scale at five minutes. and comorbidities; and random effects for contextual variables in this way: *(i)* administrative planning areas. type of health care facility. and level of care in Model 1. *(ii)* administrative planning areas. and level of care in Model 2; and *(iii)* level of care in Model 3. | | | |
