# Supplementary material for: Is a higher altitude associated with shorter survival among at-risk neonates?
Source: PLoS One. 2021 Jul 14;16(7):e0253413. doi: 10.1371/journal.pone.0253413 (PMC8279317; doi:10.1371/journal.pone.0253413)
Supplement: S4 Table — (DOCX) [file pone.0253413.s009.docx]

## S4 Table.- Crude and adjusted hazard ratios of the incidence rate of death per each altitude stratum, estimated by the final (parsimonious) mixed-effects Cox proportional hazards model, excluding neonates who died from asphyxia related disorders.

| **Altitude of the health facility where neonates were attended** | **n (%)** | **Crude hazard ratio ^a^ (95% CI)** | ***p-value*** | **Adjusted hazard ratio ^b^**  **(95% CI)** | ***p-value*** |
| --- | --- | --- | --- | --- | --- |
| *0 to <80 m (ref.)* | 1207 (53) | 1 | - | 1 | - |
| *≥80 to <2500 m* | 282 (12) | 1.04 (0.86 to 1.26) | 0.67 | 1.11 (0.89 to 1.34) | 0.35 |
| *≥2500 to <2750 m* | 123 (5) | 1.07 (0.80 to 1.44) | 0.63 | 1.24 (0.87 to 1.77) | 0.23 |
| *≥2750 m* | 678 (30) | 1.04 (0.85 to 1.28) | 0.38 | 1.27 (0.95 to 1.69) | 0.10 |
| *p for trend* | - | 1.01 (0.93 to 1.10) | 0.72 | 1.08 (0.98 to 1.19) | 0.10 |
| ^a^ Estimated hazard ratios from mixed-effects multivariate Cox proportional models. Estimated fixed effects for altitude only; and random effects for contextual variables: administrative planning areas, type of health care facility and level of care.  ^b^ Estimated hazard ratios from mixed-effects multivariate Cox proportional models. Estimated fixed effects for the next individual variables: gestational age. birth weight. Apgar scale at five minutes. and comorbidities; and random effects for contextual variables: administrative planning areas, type of health care facility and level of care. | | | | | |
