# Supplementary material for: Is a higher altitude associated with shorter survival among at-risk neonates?
Source: PLoS One. 2021 Jul 14;16(7):e0253413. doi: 10.1371/journal.pone.0253413 (PMC8279317; doi:10.1371/journal.pone.0253413)
Supplement: S5 Table — (DOCX) [file pone.0253413.s010.docx]

## S5 Table.- Crude and adjusted hazard ratios of the incidence rate of death per each altitude stratum, estimated by the final (parsimonious) fixed effects Cox proportional hazards model, excluding neonates who died from congenital malformations.

| **Altitude of the health facility where neonates were attended** | **n (%)** | **Crude hazard ratio ^a^ (95% CI)** | ***p-value*** | **Adjusted hazard ratio ^b^**  **(95% CI)** | ***p-value*** |
| --- | --- | --- | --- | --- | --- |
| *0 to <80 m (ref.)* | 1254 (54) | 1 | - | 1 | - |
| *≥80 to <2500 m* | 313 (14) | 0.96 (0.80 to 1.14) | 0.61 | 1.17 (0.96 to 1.42) | 0.11 |
| *≥2500 to <2750 m* | 127 (5) | 0.97 (0.72 to 1.31) | 0.84 | 1.22 (0.88 to 1.70) | 0.22 |
| *≥2750 m* | 624 (27) | 0.97 (0.77 to 1.24) | 0.82 | 1.25 (0.96 to 1.62) | 0.09 |
| *p for trend* | - | 0.99 (0.91 to 1.10) | 0.79 | 1.08 (0.99 to 1.18) | 0.07 |
| ^a^ Estimated hazard ratios from mixed-effects multivariate Cox proportional models. Estimated fixed effects for altitude only; and random effects for contextual variables: administrative planning areas, type of health care facility and level of care.  ^b^ Estimated hazard ratios from mixed-effects multivariate Cox proportional models. Estimated fixed effects for the next individual variables: gestational age. birth weight. Apgar scale at five minutes. and comorbidities; and random effects for contextual variables: administrative planning areas, type of health care facility and level of care. | | | | | |
