# Supplementary material for: Is a higher altitude associated with shorter survival among at-risk neonates?
Source: PLoS One. 2021 Jul 14;16(7):e0253413. doi: 10.1371/journal.pone.0253413 (PMC8279317; doi:10.1371/journal.pone.0253413)
Supplement: S11 Table — (DOCX) [file pone.0253413.s016.docx]

## S11 Table.- Neonatal mortality adjusted hazard ratios per each altitude stratum according to two mixed-effects multivariate Cox proportional hazards models in which the mean Gini coefficient at the provincial level from 2014 to 2016 is considered as a contextual variable.

| **Altitude of the health facility where neonates were attended** | **n (%)** | **Adjusted hazard ratio ^a^**  **(95% CI)** | ***p-value*** |
| --- | --- | --- | --- |
| **Model 4** |  |  |  |
| *0 to <80 m (ref.)* | 1625(54) | 1 | - |
| *≥80 to <2500 m* | 405 (13) | 1.23 (1.05t o 1.44) | <0.01 |
| *≥2500 to <2750 m* | 156 (5) | 1.39 (1.13 to 1.71) | <0.01 |
| *≥2750 m* | 830 (28) | 1.17 (1.01 to 1.36) | 0.03 |
| *p for trend* | - | 1.05 (1.00 to 1.10) | 0.03 |
| **Model 5** |  |  |  |
| *0 to <80 m (ref.)* | 1625(54) | 1 | - |
| *≥80 to <2500 m* | 405 (13) | 1.41 (1.24 to 1.61) | <0.01 |
| *≥2500 to <2750 m* | 156 (5) | 1.59 (1.32 to 1.92) | <0.01 |
| *≥2750 m* | 830 (28) | 1.22 (1.10 to 1.36) | <0.01 |
| *p for trend* | - | 1.06 (1.02 to 1.10) | 0.03 |
| ^a^ Estimated hazard ratios from mixed-effects multivariate Cox proportional models. All models (4 and 5) estimated fixed effects for the next individual variables: gestational age. birth weight. Apgar scale at five minutes. and comorbidities; and random effects for contextual variables in this way: *(i)* GINI coefficient at the provincial level, type of health care facility. and level of care in Model 4. *(ii)* GINI coefficient at the provincial level in Model 5. | | | |
