# Supplementary material for: Is a higher altitude associated with shorter survival among at-risk neonates?
Source: PLoS One. 2021 Jul 14;16(7):e0253413. doi: 10.1371/journal.pone.0253413 (PMC8279317; doi:10.1371/journal.pone.0253413)
Supplement: S12 Table — (DOCX) [file pone.0253413.s017.docx]

## S12 Table.- Neonatal mortality adjusted hazard ratios per each altitude stratum according to two mixed-effects multivariate Cox proportional hazards models in which rural-urban health care is considered as a contextual variable.

| **Altitude of the health facility where neonates were attended** | **n (%)** | **Adjusted hazard ratio ^a^**  **(95% CI)** | ***p-value*** |
| --- | --- | --- | --- |
| **Model 6** |  |  |  |
| *0 to <80 m (ref.)* | 1625(54) | 1 | - |
| *≥80 to <2500 m* | 405 (13) | 1.21 (1.06 to 1.39) | <0.01 |
| *≥2500 to <2750 m* | 156 (5) | 1.33 (1.10 to 1.62) | <0.01 |
| *≥2750 m* | 830 (28) | 1.10 (0.99 to 1.23) | 0.06 |
| *p for trend* | - | 1.04 (1.00 to 1.07) | 0.04 |
| **Model 7** |  |  |  |
| *0 to <80 m (ref.)* | 1625(54) | 1 | - |
| *≥80 to <2500 m* | 405 (13) | 1.43 (1.27 to 1.61) | <0.01 |
| *≥2500 to <2750 m* | 156 (5) | 1.61 (1.34 to 1.92) | <0.01 |
| *≥2750 m* | 830 (28) | 1.22 (1.11 to 1.35) | <0.01 |
| *p for trend* | - | 1.07 (1.04 to 1.10) | <0.01 |
| ^a^ Estimated hazard ratios from mixed-effects multivariate Cox proportional models. All models (6 and 7) estimated fixed effects for the next individual variables: gestational age. birth weight. Apgar scale at five minutes. and comorbidities; and random effects for contextual variables in this way: *(i)* Rural or urban health care, type of health care facility, and level of care in Model 6, and *(ii)* rural-urban healthcare facility in Model 7. | | | |
