# Supplementary material for: Is a higher altitude associated with shorter survival among at-risk neonates?
Source: PLoS One. 2021 Jul 14;16(7):e0253413. doi: 10.1371/journal.pone.0253413 (PMC8279317; doi:10.1371/journal.pone.0253413)
Supplement: S13 Table — (DOCX) [file pone.0253413.s018.docx]

## S13 Table.- Adjusted odds ratios of death <24h, <48h, <72h, and <7 days of life per each altitude stratum according to four mixed-effects multivariate logistic regression models adjusted by the covariates of the model 1 (see main text and table 3S).

| **Altitude of the health facility where neonates were attended** | **n (%)** | **Adjusted odds ratios^a^** | | | | | | | |
| --- | --- | --- | --- | --- | --- | --- | --- | --- | --- |
|  |  | **Death <24h of life**  **(95% CI)** | ***p-value*** | **Death <48h of life**  **(95% CI)** | ***p-value*** | **Death <72h of life**  **(95% CI)** | ***p-value*** | **Death <7 d of life**  **(95% CI)** | ***p-value*** |
| *0 to <80 m (ref.)* | 1625 (54) | 1 | - | 1 | - | 1 | - | 1 | - |
| *≥80 to <2500 m* | 405 (13) | 1.10 (0.68 to 1.76) | 0.71 | 1.26 (0.86 to 1.86) | 0.23 | 1.12 (0.76 to 1.65) | 0.56 | 1.13 (0.73 to 1.74) | 0.57 |
| *≥2500 to <2750 m* | 156 (5) | 2.28 (1.15 to 4.55) | 0.01 | 1.41 (0.77 to 2.58) | 0.26 | 1.14 (0.60 to 2.16) | 0.68 | 1.23 (0.58 to 2.61) | 0.59 |
| *≥2750 m* | 830 (28) | 1.82 (1.00 to 3.28) | 0.04 | 1.43 (0.91 to 2.24) | 0.12 | 1.49 (0.93 to 2.40) | 0.09 | 1.37 (0.77 to 2.45) | 0.28 |
| *p for trend* | - | 1.20 (0.99 to 1.46) | 0.06 | 1.13 (0.97 to 1.31) | 0.11 | 1.15 (0.97 to 1.37) | 0.10 | 1.11 (0.92 to 1.35) | 0.27 |
| ^a^ Mixed-effects multivariate logistic regression models adjusted by next individual variables: gestational age, birth weight, Apgar scale at five minutes, and comorbidities; and random effects for contextual variables: administrative planning areas, type of health care facility, and level of care. | | | | | | | | | |
