# Supplementary material for: Is a higher altitude associated with shorter survival among at-risk neonates?
Source: PLoS One. 2021 Jul 14;16(7):e0253413. doi: 10.1371/journal.pone.0253413 (PMC8279317; doi:10.1371/journal.pone.0253413)
Supplement: S14 Table — (DOCX) [file pone.0253413.s019.docx]

## S14 Table.- Adjusted estimations of survival in days of life across altitudes by Inverse-probability-weighted estimators (IPW)[5] using the covariates of the Model 1 (see main text and table 3S) at different artificial cut-offs of the follow up.

| **Altitude of the health facility where neonates were attended** | **Adjusted estimated average survival in days of life by IPW estimators^a^** | | | | | | | |
| --- | --- | --- | --- | --- | --- | --- | --- | --- |
|  | **Cut-off at 15 days of follow-up** | ***p-value*** | **Cut-off at 17 days of follow-up** | ***p-value*** | **Cut-off at 20 days of follow-up** | ***p-value*** | **Cut-off at 23 days of follow-up** | ***p-value*** |
| *<80 m* | 3.9 (3.7 to 4.1) | <0.01 | 4.3 (4.0 to 4.5) | <0.01 | 4.8 (4.5 to 5.1) | <0.01 | 5.4 (5.0 to 5.7) | <0.01 |
| *≥80 to <2500 m vs. <80m* | -0.6 (-1.0 to -0.1) | 0.01 | -0.7 (-1.2 to -0.3) | <0.01 | -1.1 (-1.6 to -0.6) | <0.01 | -1.4 (-2.0 to -0.8) | <0.01 |
| *≥2500 to <2750 m vs. <80m* | -1.2 (-1.9 to -0.5) | <0.01 | -1.3 (-2.0 to -0.6) | <0.01 | -1.3 (-2.1 to -0.5) | <0.01 | -1.7 (-2.5 to -0.8) | <0.01 |
| *≥2750 m vs. <80m* | -0.9 (-1.3 to -0.5) | <0.01 | -0.9 (-1.3 to -0.5) | <0.01 | -0.9 (-1.4 to -0.4) | <0.01 | -0.9 (-1.5 to -0.4) | <0.01 |
| **^a^** The coefficients represents the average difference on survival between each altitude stratum vs < 80 m, adjusted by variables in the Model 1 (*see main text and Table 3S*) | | | | | | | | |
